# Abdominal Decompression in Children

**DOI:** 10.1155/2012/180797

**Published:** 2012-03-22

**Authors:** J. Chiaka Ejike, Mudit Mathur

**Affiliations:** Division of Pediatric Critical Care, Department of Pediatrics, School of Medicine, Loma Linda University, Loma Linda, CA 92354, USA

## Abstract

Abdominal compartment syndrome (ACS) increases the risk for mortality in critically ill children. It occurs in association with a wide variety of medical and surgical diagnoses. Management of ACS involves recognizing the development of intra-abdominal hypertension (IAH) by intra-abdominal pressure (IAP) monitoring, treating the underlying cause, and preventing progression to ACS by lowering IAP. When ACS is already present, supporting dysfunctional organs and decreasing IAP to prevent new organ involvement become an additional focus of therapy. Medical management strategies to achieve these goals should be employed but when medical management fails, timely abdominal decompression is essential to reduce the risk of mortality. A literature review was performed to understand the role and outcomes of abdominal decompression among children with ACS. Abdominal decompression appears to have a positive effect on patient survival. However, prospective randomized studies are needed to fully understand the indications and impact of these therapies on survival in children.

## 1. Introduction

Abdominal compartment syndrome (ACS) increases the risk of mortality in critically ill children [1]. It results in 100% mortality if left untreated [2, 3]. The importance of ACS is increasingly becoming appreciated in the pediatric intensive care setting. Surgical decompressive laparotomy (DL) with open abdomen management (OA) for ACS is the definitive treatment of choice when medical and less invasive therapies have failed [4–6]. Its use has shown improvement in organ function and mortality, though mortality still remains high [7–9]. DL is an invasive therapy that is challenging to manage and often associated with significant morbidity and mortality [6, 8, 9]. Surveys have suggested that some physicians are hesitant to use DL in their patients [10, 11]. Decompression of the abdomen has also been achieved in certain cases by less invasive therapies such as catheter decompression, escharotomy, and subcutaneous fasciotomies [12–15]. This article highlights conditions associated with ACS in children and focuses on the role of abdominal decompression in its management.

## 2. Materials and Methods

The National Library of Medicine (PUBMED) was queried for “decompressive laparotomy and children”; “decompressive laparotomy and pediatrics”; “decompression laparotomy and children”; “decompression laparotomy and pediatrics”, “percutaneous drainage and abdominal compartment.” These searches yielded a total of 67 articles. Eight articles were excluded for language other than English, and 37 were not selected because they were irrelevant or exclusively related to adults. Hence, 22 articles pertinent to the role of abdominal decompression in children were included for review.

## 3. Results and Discussion

There is a relative paucity of literature on ACS in children compared to adults. Publications related to abdominal decompression in children are lacking in their scope and generalizability of findings. ACS definitions differed among the studies which could influence outcomes by unintentionally selecting patients with ACS at varying stages of progression. The majority of publications are case reports or case series, and amongst the outcome studies reviewed, none included randomization of assigned treatment. Outcomes including mortality and postoperative complications vary widely between studies and cannot be directly compared because of small numbers, varied reporting criteria, and differences in study design. The differences seen may be due to a number of contributing factors such as the populations studied; the underlying diagnoses that led to ACS; pre-existing comorbidities such as congenital malformations or genetic syndromes; timing and type of decompression; the type of temporary abdominal closure used and the grade of IAH that the patient presented with. The reported clinical experience of abdominal decompression in children is presented in Table 1. Despite the limitations discussed above, these studies illustrate the importance of early diagnosis and timely abdominal decompression in the management of ACS. The adverse physiological effects of IAH start long before the manifestation of ACS becomes clinically evident [20]. Therefore, the question of appropriate timing for decompression is a pressing issue that remains unclear. Overall outcomes are also affected by the techniques used to decompress the abdomen, and the materials and management strategies applied to manage the OA and support dysfunctional failing organs that accompany the diagnosis of ACS. 

Definitions related to IAH and ACS in children are derived from the consensus statements put forward by the WSACS (World Society of Abdominal compartment Syndrome; http://www.wsacs.org). However, these definitions are not all directly applicable in children and suggested definitions specific to children have been published and are presented in Table 2 [21].

The true incidence of ACS in children is difficult to determine because of the wide variety of illnesses it is associated with, the relative lack of consensus on the threshold IAP used to define ACS in children, and the paucity of publications. Studies have reported occurrence rates between 0.6 and 9.8% in single-center studies involving critically ill children [1, 7, 8, 17, 22].

### 3.1. Risk Factors and Conditions Associated with ACS in Children

Risk factors that predispose patients to IAH and ACS can be grouped into four major categories based on the pathophysiology of elevated IAP.

1. *Decreased Abdominal Wall Compliance.* circumferential burns involving the abdomen, constrictive dressings, or tight closure of the abdomen following abdominal surgery can lead to decreased abdominal wall compliance.
2. *Increased Intraluminal Contents.* the accumulation of air, stool, or fluid in the intestines as can be seen in Hirschprung's disease, toxic megacolon, or ileus can lead to elevations in IAP resulting in ACS.
3. *Increased Intra-Abdominal Contents.* intra-abdominal space-occupying lesions such as tumors, intraperitoneal fluid, abscesses, and intra-abdominal hemorrhages can result in ACS.
4. *Capillary Leak Syndrome/Fluid Resuscitation*. aggressive fluid resuscitation especially with crystalloid solutions in the critically ill or diseases associated with capillary leak syndrome can lead to the development of secondary ACS.

Some conditions reported to be associated with ACS in children are listed in Table 3.

There may be an increased risk for the development of ACS among children with congenital malformations and genetic disorders. In Steinau's series, 12 out of 26 children who developed ACS had at least one underlying congenital malformation or genetic disorder [9]. In two other pediatric studies, congenital malformations (included arthrogryposis, caudal regression syndrome, biliary atresia, and cystic fibrosis) were seen in 20% (*n* = 2) of children with ACS [7, 17].

The WSACS recommends that IAP measurement be obtained if two or more risk factors for IAH/ACS are present. If IAH is detected, serial IAP measurements should be performed [4, 5].

### 3.2. IAP Measurement in Children

Techniques used to measure IAP can be divided into two groups, the direct and the indirect method. The direct method entails placing a needle or catheter directly into the peritoneal space and transducing the pressure in the abdomen. In clinical practice, its use solely for IAP measurement is limited by the potential for complications such as bowel perforation and peritoneal contamination [23]. However, direct IAP measurement can be easily performed when a peritoneal catheter is already in place or the placement of one is indicated as a therapeutic measure. Indirect methods measure IAP via routes other than the peritoneal space. The intravesical method is the gold standard for measuring IAP [23–25]. Several surveys indicate that it is currently the most commonly used technique in children and adults [10, 11, 26]. IAP is measured via a urethral catheter placed in the bladder. A debubbled fluid column is created by infusing sterile normal saline into the bladder. The optimum volume to use in children weighing 2.7 to 50 kg is 3 mL or 1 mL/kg up to a maximum of 25 mL [19]. In children weighing more than 50 kg, 25 mL can be used as the instillation volume as recommended by the WSACS for adults [4, 5]. Appropriate volumes for infants weighing less than 2.7 kg have not been determined. For accurate measurements using this method, the transducer is zeroed at the mid-axillary line with the patient in the supine position. IAP should be recorded at end expiration and when the abdominal muscles are relaxed. A period of 30–60 seconds should be allowed for equilibration of the pressure within the bladder before recording IAP [4, 5]. Other indirect methods reported in the literature include the intragastric, intrarectal, intrauterine, and venacaval methods [23, 25, 27].

### 3.3. Management of IAH and ACS

The main goals in management of IAH include recognizing its presence by objective IAP monitoring, treating the underlying cause, and preventing progression to ACS by lowering IAP.

 Medical management strategies to lower IAP and improve organ perfusion can be guided by the following principles [4].

1. Evacuate intraluminal contents via nasogastric or rectal tubes; avoiding or minimizing enteral feeds; administering enemas or prokinetic agents; colonoscopic decompression.
2. Improve abdominal wall compliance by ensuring adequate sedation, analgesia, and neuromuscular blockade; removal of constrictive dressings and placing in the reverse Trendelenberg position.
3. Optimize fluid administration by avoiding excessive fluid resuscitation (especially with crystalloids, consider using hypertonic fluids and colloids early); aiming for a net negative or zero fluid balance by the third day from initial resuscitation and begin fluid removal through judicious diuresis, ultrafiltration, or dialysis once stable.
4. Optimize systemic and regional perfusion by goal directed fluid resuscitation, hemodynamic monitoring to guide resuscitation, and use of vasoactive medications to maintain adequate abdominal perfusion pressures.
5. Evacuate free intraperitoneal fluid or air, by paracentesis or percutaneous catheter drainage.

Identification and specific treatment of the underlying cause of IAH must be addressed concomitantly. Abdominal ultrasound or Computerized Tomography can be useful in diagnosing the underlying cause and directing management. If the underlying cause of IAH or ACS is surgical, medical management strategies should be used to stabilize the patient without delaying definitive surgery. When medical management is failing and ACS is already present, surgical DL should be performed promptly.

### 3.4. Decompressive Laparotomy

Decompressive laparotomy (DL) is essential and potentially lifesaving in treating IAH-induced organ failure [4]. It has resulted in improvement in physiologic parameters associated with ACS and mortality. Beck et al. retrospectively studied 10 patients with 15 episodes of ACS. They defined ACS as increasing abdominal distention with IAP of >15 mmHg, accompanied by at least three of the following major criteria: oliguria (urine output < 1 mL/kg/hr) or anuria, refractory to volume expanders or diuretics; hemodynamic instability or hypotension refractory to volume expanders; reduced chest compliance leading to increasing PaCO_2_ and decreasing PaO_2_/FiO_2_ ratio and requiring higher FiO_2_ and ventilatory pressures; metabolic acidosis with a base deficit >6 mMol/L. Physiologic parameters were compared 4 hrs before ACS development and 4 hours after abdominal decompression. Mean arterial pressure, PaO_2_, PaO_2_/FiO_2_ ratio, and urinary output decreased significantly, whereas PaCO_2_, peak inspiratory pressures (PIPs), positive end-expiratory pressures (PEEPs), and base deficit increased significantly after the development of ACS. After DL, these variables returned to pre-ACS values. Overall mortality rate in this group was 60%. Pearson et al. studied 26 children with ACS who required emergency laparotomy. They defined ACS as sustained bladder pressure >12 mmHg that was associated with new onset organ dysfunction or failure. They demonstrated improvement of the following physiologic parameters: need for fluid resuscitation, oxygen index, mean airway pressure, vasopressor score, but urine output continued to be minimal 12 hours after DL. Overall mortality was 58% in this cohort [8]. Neville et al. demonstrated decreased oxygen requirements after patch abdominoplasty performed for clinical deterioration associated with criteria defining ACS. PIP decreased also but only in ACS survivors, and oxygen requirements decreased more significantly in survivors compared to nonsurvivors in this cohort. They defined ACS as an increase oxygen requirement and elevation in PIP associated with abdominal distention, worsening renal and cardiac function. The mortality in this cohort was 34.8% [18].

DL is the treatment of choice for most patients with IAH or ACS that is refractory to nonoperative medical management strategies and it is associated with significantly improved patient survival [68, 69]. It needs to be performed before irreversible organ dysfunction occurs for better outcomes. Steinau et al. retrospectively reviewed outcomes of ACS in children using a therapeutic algorithm that focused on timely decompression. They showed a survival rate of 78.6% [9]. This is the best survival rate of ACS reported in children thus far, though their approach to abdominal decompression was proactive with IAP > 12 mmHg and only one organ dysfunction constituting triggers for DL. Optimization of conservative therapies and early decompression guided by use of a therapeutic algorithm likely contributed to improved outcomes. Cheatham and Safcsak have shown that using an evidenced based management algorithm in adult patients improved patient survival to discharge from 50 to 75%, and rates of primary fascial closure from 59 to 81% [69].

DL involves making an incision to open all layers of skin, subcutaneous tissue, fascia, and peritoneum [6]. Both midline and transverse incisions have been used in children [8, 9, 51]. Ideally, DL should be performed in the operating room. In situations where the patient is too unstable for transport or if an operating room is not readily available, DL can be performed in the pediatric intensive care unit (PICU). In Pearson et al.'s report 13 of 27 patients had DL performed at the bedside and 4 of 15 episodes of ACS in Beck et al.'s study had DL performed at the bedside [7, 8]. Successful management of ACS using DL may depend upon the use of an open abdomen management strategy.

### 3.5. Open Abdomen Management

DL is often supported by open abdomen (OA) management to avoid recurrence of ACS. OA management is achieved by leaving the fascia and the skin open, and temporarily covering the viscera. An OA with temporary abdominal closure (TAC) may also be necessary following operations in which edematous viscera preclude easy fascial closure, management of abdominal wall defects or in which an adult size organ has been transplanted into a small child [22, 34, 52, 53, 55, 70, 71]. The ultimate goal of OA management is to achieve prompt primary fascial closure without complications. To achieve successful primary fascial closure entails a multidisciplinary approach in management of the open abdomen [72]. The ICU team is working toward safely removing excess fluid while preserving organ function, preventing infection, and treating concurrent comorbidities. The surgical team has the task of choosing the ideal TAC technique for each patient and managing dressing changes. To avoid the development of recurrent ACS and lateral retraction of the abdominal muscles, the surgical team should constantly be seeking out the earliest optimal time for primary fascial closure. The challenge of OA management increases when delayed primary fascial closure is not achievable because abdominal wall reconstruction will need to be addressed at a later date [55]. Most pediatric studies reviewed did not focus on successful achievement of primary fascial closure after management of ACS. Three studies addressed it. A study by Steinau et al. reports 72% of patients achieved primary fascial closure in a median of 53 (range 10–63) days. Incisional hernias needing repair occurred in 27.3%, one needed closure of a stoma and another one needed relaparotomy for an ileus due to adhesions [9]. DL with OA management was used in 26 patients with ACS. Time to definitive abdominal closure averaged 8.6 days (range, 1–61 days) needing an average of 3.2 separate operations (range, 1–14) to achieve abdominal wall closure. This study did not clarify if primary fascial closure was achieved or not. Postoperative complications that could be related to DL in this study included wound infection (*n* = 5), fascial dehiscence (*n* = 1), and enterocutaneous fistula (*n* = 1) [8]. In another study, 11 of 23 (47.8%) patients who underwent patch abdominoplasty for ACS achieved delayed primary fascial closure. Complications in this cohort included mesh infection (*n* = 2), recurrent ACS (*n* = 1), and subsequent incisional hernia for the five patients that did not achieve primary fascial closure [18].

### 3.6. Temporary Abdominal Closure

Temporary abdominal closure (TAC) techniques have recently been characterized into three generations reflecting the historical evolution of the devices, from simple coverings, to contain the viscera, to closure devices to cover and aid in the gradual approximation of the abdominal edges [9, 42, 70, 72, 73]. The first generation aimed at bridging the defect and covering the abdomen, using biodegradable or synthetic materials. The second generation aimed at fluid control and improved barrier function and the third generation aimed at gradual approximation of the wound by the use of gradual manual tension such as the Wittmann patch or negative pressure [72]. Several of these techniques have been reported in children and are presented in Table 4.

### 3.7. Catheter Decompression

Abdominal decompression by catheter decompression is gaining favor because of its less invasive quality and decreased morbidity associated with its use. Abdominal decompression has been achieved by percutaneous catheter decompression in situations in which intra-abdominal fluid plays a significant role in the underlying diagnosis [14, 31, 37, 49]. Bedsides, sonography can assist in distinguishing patients with progressive IAH predominately related to intraperitoneal fluid, from those with bowel wall edema or a space-occupying lesion, where catheter decompression will not be useful and may lead to unnecessary complications.

Percutaneous drainage (PD) of peritoneal fluid can prevent the progression of IAH to ACS altogether or may allow time to stabilize the patient for DL [14, 31, 49, 50]. Improvement in physiologic criteria has been seen with insertion of PD for IAH and ACS. A retrospective review was performed of 32 neonates who had IAP monitoring. Clinical presentation was divided into I and II grade signs. I grade signs included increased abdominal circumference, reduced abdominal wall compliance, and ultrasound confirmation of fluid in the abdominal cavity. II grade signs included necrotizing enterocolitis, septic shock, renal failure, low cardiac output, increased central venous pressure, delayed capillary refill time, acute respiratory failure, increased PaCO_2_, decreased PaO_2_, oliguria/anuria, and weak or absent femoral artery pulses. Twenty-eight patients met criteria for IAH, defined as IAP > 10 mmHg with each I grade sign and two symptoms from II grade signs while ACS was defined as IAP > 20 mmHg, with each I grade sign plus at least three symptoms of grade II signs. Insertion of a PD was performed in all cases. After insertion, resolution of symptoms was observed in all patients but with varying improvements in clinical parameters and measurements [12]. A study including 10 PICU patients with ACS reported a decrease in IAP and ventilatory parameters and an increase in urine output rapidly after abdominal decompression performed by PD in 5 patients and DL in 2 patients. The two patients with DL subsequently died while only one of the five with PD died. Overall mortality in this study was 40%. Latenser et al. in a pilot study compared PD with DL in adult and pediatric patients with greater than 40% total body surface area (TBSA) burns. There was reduction in IAP after catheter placement in all nine patients with IAH. Only 4 patients with failed PD required emergent DL after developing ACS. They concluded that PD is safe and effective as a decompression modality for decreasing IAH and preventing ACS in patients with less than 80% TBSA burns [14]. It can be very useful in patients where DL is less desirable because of a high surgical risk. PD successfully avoided the need to perform a DL in an anticoagulated-patient undergoing extracorporeal life support who developed ACS [37].

Paracentesis using angiocatheters, peritoneal dialysis catheters, and hemodialysis catheters have been reported [14, 31, 49, 74]. Needle decompression was reported in a child with ACS resulting from tension pneumoperitoneum [45]. The successful use of a Penrose drain has been reported in a very-low-birth-weight (VLBW) neonate weighing only 650 gm [75].

Complications with catheter decompression have not been well reported in the literature. Latenser et al.'s study reported peritoneal contamination that did not progress to peritonitis in one patient [14]. The VLBW neonate developed hernias at the catheter insertion sites upon removal of the Penrose drain, resulting in the need for reconstructive laparoscopic surgery [75]. Some patients have progressed to death in spite of decompression by catheter placement [49]. It is important to note that when IAH or ACS is not ameliorated by catheter decompression and aggressive medical management, DL should be performed immediately [74].

DL was widely considered the only therapeutic option for abdominal decompression in the recent past but less invasive methods of abdominal decompression like catheter decompression are gaining favor because of the decreased morbidities they may offer compared to DL with OA management. Only patients with moderate amounts of free intraperitoneal fluid and no surgical intra-abdominal pathology may benefit from this procedure. Patients with no surgical intra-abdominal pathology and insignificant amounts of intraperitoneal fluid will need other means of decompression. Minimally invasive subcutaneous fasciotomy has been described in adults but its use has not been reported in pediatrics. It entails subcutaneous division of the linea alba between cutaneous incisions or subcutaneous anterior medial rectus abdominis fasciotomy through small skin incisions (with drainage of intra-abdominal fluid) [76, 77].

Reported complications with OA management include intra-abdominal abscesses, enteroatmospheric fistulae, and herniae when primary fascial closure cannot be achieved. It is difficult to tease out morbidity or mortality associated with DL or OA management from morbidity and mortality related to the underlying cause and the progression of ACS itself. However, arguments for its benefits can be made based on the improvements in physiologic parameters and when death from ACS is imminent, in its absence [78]. Large randomized studies could help answer this question but based on the few publications that suggest benefit with its use, it might become ethically challenging not to provide DL in the control group when the need arises. Few studies have looked at long-term complications from DL and OA management. A questionnaire was sent to the parents of 22 ACS-survivors following DL with a resulting response rate of 72.7% (16/22). According to the responses to the questionnaire, the scar area brought physiological or psychological discomfort to 10 of 16 children. Of those, subjective impediment degree was put at 100% by five children, at 80% by one child, and at 50% by two [9].

An adult study looking at the long-term impact of DL on physical and mental health, quality of life, and subsequent employment showed that DL initially decreased physical, but not mental health perception compared with that of the United States general population, and that abdominal wall reconstruction restores physical and mental health to normal levels [79]. Another similar but prospective study amongst adults compared patients discharged with an incisional hernia, and primary fascial closure after DL, to the general public. It demonstrated that at 6 months postdecompression, physical and social functions were significantly decreased among the incisional hernia group but not the primary fascial closure group. By 18 months, the incisional hernia group had normal physical and mental health. The two groups had decreased but similar quality-adjusted life years and similar ability to resume employment. They concluded that ACS does not appear to have a long-term impact on physical or mental health, whether same admission primary fascial closure is possible or a chronic incisional hernia is required. ACS is not as debilitating and life altering as might be expected [80].

## 4. Conclusions

ACS is a potentially lethal condition associated with a wide variety of conditions some of which are seemingly innocent at presentation. Clinicians should screen children in the intensive care unit at risk for developing ACS on an ongoing basis. ACS must be recognized early. Clear consensus definitions specific to pediatric patients based on organized studies are still necessary and will help in ACS recognition. Appropriate treatment options supporting organ function and timely assessment for catheter or surgical decompression should be employed. Current therapies being utilized for abdominal decompression appear to have a positive impact on patient survival. However, prospective randomized studies are needed, to fully understand the indications and impact of therapies directed at IAH and ACS management on survival in children. A number of children who would have died without intervention may survive after management of ACS by abdominal decompression, but may acquire varying physiological and psychological complications. Long-term studies to understand the impact of abdominal decompression techniques need to be conducted in children.

## Figures and Tables

**Table 1:** Reported clinical experience with abdominal decompression in children.

Study (Year)	Study type	Population	*n*	ACS definition used	ACS incidence (%)	Mortality (%)	Decompression type	1° closure fascial (%)	Days to closure	Complications of decompression
Akhobadze et al. (2011) [12]	R	Neonates with IAP monitoring	32	IAP > 20 mmHg + 3 specified SOD	34	Grade I and II —17 Grade III —37.5 Grade IV —100	PD	NA	NA	None

Steinau et al. (2011) [9]	R	Neonates and children with ACS	28	IAP > 12 mmHg + 1 specified SOD	NA	21.4	DL	64.2	53 (10–63)	ECF-21.4%, hernia needing repair-27.3%

Pearson et al. (2010) [8]	R	Children with exploratory laparotomy	264	IAP > 12 mmHg + new SOD	9.8	58	DL	100.0	8.6 (1–61)	ECF, Renal failure

Ejike et al. (2007) [1]	P	Critically ill children with mechanical ventilation	75	IAP > 12 mmHg with new SOD	4.7	ACS –50 non-ACS –8.1	PD, DL	NR	NR	ECF

Hershberger et al. (2007) [16]	R	Burn patients (adults and children); 7 children (ages 6 months to 8 years)	25 of 5195	IAP > 12 mmHg + specified SOD	NR	88	DL (*n* = 25), truncal escharaotomy in addition to DL (*n* = 13)	16.0	NR	NR

Diaz et al. (2006) [17]	P	Critically ill children	1052	IAP > 10 mmHg + SOD	0.9	40	DL (*n* = 2) or PD (*n* = 5)	NR	NR	NR

Latenser et al. (2002) [14]	P	Burn patients (adults and children) with >40% TBSA burns	9	≥30 mm Hg + pulmonary or renal dysfunction	0.7	No IAH —50, IAH with PD—40, DL—100	PD, DL with chest/abdominal escharotomy	NR	NR	NR

Beck et al. (2001) [7]	P	PICU patients	1762	abdominal distention + IAP > 15 mmHg + at least 2 SOD	0.6 (0.7% of trauma pts)	60	DL with Dacron Mesh or Bogota bag	NR	NR	Recurrent ACS

Neville et al. (2000) [18]	R	Patch abdominoplasty for ACS	23	Elevated PIP, O_2_ req., or worsening renal or cardiac function	NR	34.7	DL with patch abdominoplasty	47.8%	6 (2–11)	Intra-abdominal abscess and ECF

IAH, intra-abdominal hypertension; ACS, abdominal compartment syndrome; IAP, intra-abdominal pressure; *n*, number of patients, 1°, primary; R, retrospective; P, prospective; SOD, signs of organ dysfunction; PD, peritoneal dialysis; DL, decompressive laparotomy; PICU, Pediatric intensive care unit; ECF, enterocutaneous fistula; TBSA, total body surface area; NR, not reported, PIP, peak inspiratory pressure; req, requirement.

Adult studies that included pediatric patients.

**Table 2:** The WSACS consensus definitions and suggested pediatric definitions.

	WSACS consensus definitions [4, 5]	Suggested pediatric definitions
IAP	The pressure concealed within the abdominal cavity (It should be expressed in mmHg and measured at end expiration)	*Same*

Normal IAP	Approximately 5–7 mmHg in critically ill adults	7 ± 3 mmHg in critically ill children [19]

APP	The difference between MAP and IAP	*Same*

IAH	Defined by a sustained or repeated pathological elevation in IAP ≥ 12 mmHg.	Defined by a sustained or repeated pathological elevation in IAP ≥ 10 mmHg [19].

IAH grade I	IAP 12–15 mmHg	IAP 10–12 mmHg

IAH grade II	IAP 16–20 mmHg	IAP 13–15 mmHg

IAH grade III	IAP 21–25 mmHg	IAP 16–19 mmHg

IAH grade IV	IAP *> *25 mmHg	IAP ≥ 20 mmHg

ACS	Sustained IAP *> *20 mmHg (with or without an APP *< *60 mmHg) that is associated with new organ dysfunction/failure	A sustained IAP of greater than 10 mmHg associated with new organ dysfunction/failure

Primary ACS	A condition associated with injury or disease in the abdomino-pelvic region that frequently requires early surgical or interventional radiological intervention	*Same*

Secondary ACS	Refers to conditions that do not originate from the abdomino-pelvic region	*Same*

Recurrent ACS	Refers to the condition in which ACS redevelops following previous surgical or medical treatment of primary or secondary ACS	*Same*

WSACS, World Society of Abdominal Compartment Syndrome; IAP, intra-abdominal pressure; IAH, intra-abdominal hypertension; APP, abdominal perfusion pressure; MAP, mean arterial pressure; ACS, abdominal compartment syndrome.

**Table 3:** Reported conditions associated with ACS in children.

Primary ACS	
*Decreased abdominal wall compliance*	
Gastroschisis [28]	
Cantrell Syndrome [29]	
*Increased intraluminal contents*	
Small intestine intussusception [30]	
Ileus [9]	
Hirschprung's disease [18]	
*Increased abdominal contents*	
Intra-abdominal trauma (edematous viscera) [9, 18, 31–33]	
Intestinal transplantation [22, 34, 35]	
Intra-abdominal bleeding/retroperitoneal bleeding [9, 36]	
GI bleeding	
Extracorporeal life support [37–39]	
Nonpancreatic pseudocyst [40]	
Wilm's Tumor [18, 41]	
Neuroblastoma [42, 43]	
Burkitt's Lymphoma [1]	
Pyonephrosis/obstructive megaureter [44]	
Pancreatitis [9]	
Tension pneumoperitoneum/intestinal perforation [45]	
Peritonitis/intra-abdominal infection [1, 9]	
Infectious enterocolitis [8, 18]	
Post surgical complication (abdominal surgery) [8, 18]	
Bowel obstruction or perforation [1, 9, 18]	

Secondary ACS	

*Capillary leak/fluid resuscitation*	
Sepsis/Septic shock [1, 9, 37, 46]	
Toxic shock syndrome [1]	
Dengue shock syndrome [47]	
Trauma shock [48]	
Cardiogenic shock/cardiac arrest [1]	
Burns [9, 14, 16, 49, 50]	

ACS, abdominal compartment syndrome.

**Table 4:** Temporary abdominal closure techniques reported in children.

Study	*n*	TAC	Primary fascial closure (%)	Complications	Conclusion
Keene et al. (2011) [42]	2	Prolene mesh (Pt 1); Extracellular matrix mesh and vacuum therapy (Pt 2)	0.0	Patient 1-sepsis and ECF; Patient 2-skin dehiscence, infected extracellular mesh. Both patients healed by secondary intention	Using extracellular matrix mesh and vacuum therapy for fascial and skin closure, respectively, is superior to Prolene mesh

Biebl et al. (2010) [51]	5	Neuropatches (3), Polytetrafluoroethylene (1), Silastic sheet (1)	80.0	Subileus in one pt at 18 months post closure	Recommend early operation for ACS using patch abdominoplasty

Pentlow et al. (2008) [52]	5	Porcine dermal collagen implants	100.0	Incisional hernia, skin dehiscence over implant	Porcine dermal collagen implant is a helpful adjunct to abdominal wall closure following organ transplantation

Fenton et al. (2007) [53]	7	Temporary abdominal vacuum packing	100.0	None	Vac-Pac closure in infants is a safe and effective method of TAC

Barker et al. (2007) [54]	258*	Temporary vacuum pack	68.1	Fistulae (5%), abscesses (3.5%), bowel obstr (1.2%), ACS (1.2%), evisceration (0.4%)	Method demonstrates ease of mastery, effectiveness in patient care and comfort, low cost, and complication rates

Howdieshell et al. (2004) [55]	88*	Silicone sheeting TAC	81.0 (of survivors)	Revision of sheeting due to recurrent ACS or fascial-sheeting dehiscence	Provides a safe and reliable TAC allowing for later definitive reconstruction

Wu et al. (2003) [56]	15	Primary Silastic spring-loaded silo	100.0	Temporary dislodgement of silo (13.3%)	Permits safe, gentle, and gradual reduction of the exposed viscera

Markley et al. (2002) [57]	6	Pediatric vacuum packing wound closure and corset-like lacing	80.0	none	The Vac-Pac wound closure technique and its corset modification are important additions to the armamentarium of the general and pediatric surgeon for the management of the ACS

Tremblay et al. (2001) [58]	181*	Skin only closure, Silo, Polygalactin mesh or packing	52	ACS (13%), ECF (14%), evisceration/dehiscence (5%), hernias (48%)	No definite conclusions. Recommended prospective trials to determine the optimal technique for abdominal closure

Barker et al. (2000) [59]	112*	Temporary vacuum pack	55.4	ECF (4.5%), abscesses (4.5%), required re-exploration after closure (2.7%)	The technique is simple and easily mastered and primary closure is achieved in the majority with a low complication rate

Neville et al. (2000) [18]	23	Patch abdominoplasty	43.4	21.7%-ECF, abscesses	Patch abdominoplasty effectively decreases airway pressures and oxygen requirements associated with ACS

de Ville de Goyet et al. (1998) [60]	329*	Temporary Silastic prosthetic closure with skin closure	76.5 (36 of 47)	None related to TAC	Very useful variation of TAC that is free of related complications and esthetically preferable to others

Sherck et al. (1998) [61]	50*	Sutureless coverage (clear plastic sheet + sump drains + iodophore impregnated adhesive plastic drape)	87.5	No recurrent ACS, evisceration, wound infection, fasciitis nor bowel obstruction; ECF (2), pelvic/abdominal abscess (3), pancreatic fistula (1)	Rapid, safe, easily available means of managing the OA

Smith et al. (1997) [62]	93*	Vacuum pack	73.9 (of survivors)	ECF (4.3%), abcesses (4.3%)	Good patient outcomes can be achieved with its use and careful subsequent management

Ong et al. (1996) [63]	21	Temporary Silastic patch closure	100.0	23.8 wound complications (dehiscence = 1, infection = 3, incisional hernia = 1)	In patients with difficult abdominal closure after liver transplant recommended as treatment of choice at that time

Seaman et al. (1996) [64]	17	Polytetrafluoroethylene patch + abdominal drains with suction	100.0 (skin closure by secondary intention)	None	Suggests that PTFE can be used safely for temporary wound closure in liver transplant recipients. The majority of patches can be removed during the first postoperative week

Brock et al. (1995) [65]	28*	Vacuum pack	50.0	ECF (4), wound dehiscence (2)	Inexpensive, readily available and valuable

Shun et al. (1992) [66]	2	Expanded Polytetrafluoroethylene	100.0		Technique allows greater flexibility in use of donor livers for pediatric patients

Schnaufer and Everett (1975) [67]	2	Silastic patch	50.0	Sepsis	Can be useful in pts with stage IV neuroblastoma.

TAC, temporary abdominal closure; ACS, abdominal compartment syndrome; ECF, enterocutaneous fistula; obstr, obstruction; PTFE, polytetrafluoroethylene; OA, open abdomen.
